# Prevalence and characteristics of children with cerebral palsy according to socioeconomic status of areas of residence in a French department

**DOI:** 10.1371/journal.pone.0268108

**Published:** 2022-05-19

**Authors:** Malika Delobel-Ayoub, Virginie Ehlinger, Dana Klapouszczak, Carine Duffaut, Catherine Arnaud, Mariane Sentenac

**Affiliations:** 1 CERPOP, UMR1295 Toulouse University, Inserm, Paul Sabatier University, Toulouse, France; 2 CHU Toulouse, Registre des Handicaps de l’Enfant en Haute-Garonne, Toulouse, France; 3 Clinical Epidemiology Unit, University Hospital, Toulouse, France; 4 Université Paris Cité, Inserm, INRAE, Centre for Research in Epidemiology and StatisticS (CRESS), Obstetrical Perinatal and Pediatric Epidemiology Research Team, EPOPé, Paris, France; Yamaguchi University: Yamaguchi Daigaku, JAPAN

## Abstract

**Aim:**

To study the association between the socioeconomic environment of area of residence and prevalence and characteristics of children with cerebral palsy (CP).

**Method:**

Data on 8-year-old children with CP born in 2000–2011 (n = 252) were extracted from a regional population-based register in France. The European Deprivation Index (EDI), available at census block level, characterised socioeconomic deprivation in the child’s area of residence at age of registration. The prevalence of CP was estimated in each group of census units defined by EDI distribution tertiles in the general population. The association between deprivation level and CP severity was assessed according to term/preterm status.

**Results:**

CP prevalence differed between deprivation risk groups showing a **J**-shaped form with the prevalence in the most deprived tertile (T3) being the highest but not significantly different of the prevalence in the least deprived one (T1). However, the prevalence in the medium deprivation tertile (T2) was significantly lower than that in the most deprived one with a prevalence risk ratio (PRR) of: PRR_T2/T3_ = 0.63 _95% CI_ [0.44–0.89]). Prevalences of CP with associated intellectual disability (ID) and CP with inability to walk were significantly higher in the most deprived tertile compared to the least deprived one (respectively PRR_T3/T1_ = 1.86 _95% CI_ [1.19–2.92] and PRR_T3/T1_ = 1.90 _95% CI_ [1.07–3.37]). Compared to children living in the least deprived areas, children with CP born preterm living in the most deprived areas had more severe forms of motor impairment, such as an inability to walk or a combination of an inability to walk and moderate to severe impairment of bimanual function. They also had more associated intellectual disability. No associations were observed among term-born children.

**Interpretation:**

A significant association between area deprivation group and CP severity was observed among preterm children but not among term-born children.

## Introduction

Numerous publications tend to show that the risk of cerebral palsy (CP) increases with greater socioeconomic disadvantage [1–6] or lower parental education [7]. The mechanisms involved in the association between higher CP risk and deprivation potentially differ for post-neonatally and pre/perinatally-acquired CP. This justifies studying post- and pre/perinatally-acquired CP separately which is not done in some previous studies [5,7–10]. For children with pre/perinatally acquired CP, the well-established socioeconomic gradient in preterm birth and low birthweight [11] is presented as part of the causal pathway. However, some studies showed that these perinatal risk factors do not fully explain the excess risk for developing CP that is associated with socioeconomic disadvantage [3,5,8]. The underlying mechanisms appear to be complex and we may assume that socioeconomic background affects access to care and resources. In some studies, socioeconomic disadvantage was particularly associated with an increased risk of spastic forms of CP only [1,2,8] and in some cases a socioeconomic gradient was shown only in term-born children [1–3]. Several studies also suggested that major functional limitations among children with CP were associated with higher deprivation [1,2,10,12,13], and one of these studies [10] showed that the relationship between deprivation and functional severity of CP was higher in preterm than in full-term children. Dolk et al. [1] suggested that the deprivation-risk gradient may be greater in children with severe intellectual disabilities.

The indicators used to assess family socioeconomic deprivation vary between studies, including individual measures [2,7,9] and ecologic area measures [1,3,14,15] sometimes both in the same study [5,8,10,13]. Although correlated, these indicators reflect different and complementary mechanisms involved in the association between deprivation and disability [16].

In the present study, we analysed the association between socioeconomic disadvantage and CP risk in terms of prevalence and of functional outcomes. We hypothesize that deprivation could have an impact on the risk of developing CP and on the functional severity of CP. Two kinds of mechanisms could coexist: 1) a link between deprivation and the origin of CP which could lead to both an increase in the prevalence and a greater severity of the disorder and 2) the association between deprivation and access to some intensive, ultra-early medical and re-educational care modalities which could modify the functional prognosis of some associated disorders such as intellectual disability or fine motor skills. Given the known differences between full-term and preterm children in the origins of CP, in the type of brain damage associated with CP [17,18] and in the degree of brain maturity, we can expect that the impact of deprivation on the functional outcomes is potentially different in these two groups.

We used data from a regional child disability population-based register in France to investigate the link between socioeconomic deprivation of parents’ area of residence and CP prevalence. We then analysed how the clinical severity characteristics of CP varied with socioeconomic deprivation in term and preterm children separately.

## Methods

### Participants

Data were obtained from the childhood disability register of the department of Haute-Garonne, south-western France. This register is approved by the French National Commission for Data Protection and Liberties (CNIL) for all its usual activities and received specific authorization for using mailing addresses to locate each child in a census block. Parental consent was sought prior to inclusion in the register. Children born between 2000 and 2011 were included if they were residents of the surveillance area in the calendar year they reached the age of 8 and if they met the definition of CP developed by the SCPE network [19] of which the register is a member. CP is a group of permanent disorders involving movement, posture and motor function due to a non-progressive interference, lesion or abnormality of the developing brain. This definition excludes progressive disorders and isolated hypotonia. The diagnosis is made solely on the basis of clinical description and additional features such as imaging or laboratory results are not part of the inclusion criteria. The main data source was the local public authority centralizing all demands regarding financial allowances or educational support with regular or specialized schooling for children having widely varying disabilities. Financial and educational support are attributed independently from each other and are available to children with all degrees of CP severity, even the mild forms that benefit only from educational support at regular school. Other sources of data were the medical records of the regional hospital centre that holds the neuropediatric reference centre in the register’s coverage area. Inclusion in the register was determined by a register’s physician after a comprehensive review of all clinical records available in all data sources. Because the number of post-neonatally acquired CP was too small to be studied separately, analysis was restricted to pre/perinatal CP. A total of 257 children were eligible. Parental address at inclusion was used to geolocate each child in one of the 850 census units of the surveillance area. Geolocation was not possible for 5 children, yielding a final sample of 252 children.

### Clinical characteristics

In accordance with the SCPE network guidelines, the classification of CP subtypes is based on clinical features and made on the basis of the predominant neurological finding. The classification tree for subtypes of CP is used as reference and cases are recorded in the register according to the following categories: spastic bilateral, spastic unilateral, dyskinetic (including dystonic and choreo-athetotic) and ataxic. An "unable to classify" category includes cases for which there is insufficient information to determine the predominant neurological form. The other following characteristics of children with CP were considered: sex, preterm birth (defined as birth before 37 weeks of gestation, yes/no), low birthweight (birthweight <2500 g, yes/no) and maternal age at birth (<25 years or >38 years corresponding respectively to the 10th percentile and the 90th percentile of the sample’s distribution). The severity of CP and the associated disorders were assessed by: 1) inability to walk (defined using the Gross Motor Function Classification System (GMFCS) categories IV and V versus children who were able to walk, even with assistance, corresponding to GMFCS I to III), 2) Bimanual Fine Motor Function classification (BFMF): moderate to severe categories III to V versus BFMF I and II, 3) combination of inability to walk and moderate to severe BFMF impairment (indicator of severity of combination of manual and gross motor function independently of predominant neurologic pattern), 4) intellectual disability (ID) (defined as Intellectual Quotient <70), 5) epilepsy, 6) visual impairment (severe with acuity less than 3/10 or less severe with nystagmus or amblyopia or strabismus or other congenital anomalies) or hearing impairment (regardless of severity) and 7) the presence of a malformation (cerebral, cardiac or other location). In addition, MRI brain imaging results are presented for children born in 2004 and later only and were classified into 5 main categories defined according to the classification developed and validated by the SCPE network [20]: maldevelopments, predominant white matter injury, predominant grey matter injury, miscellaneous and normal. All imaging findings were reviewed by SCPE network experts to ensure proper and reliable classification of results.

### Socioeconomic deprivation measures

We used the French version of the European Index of Deprivation (EDI) [21], an ecological deprivation index composed of a combination of 10 indicators available at census block level (overcrowding, no access to a system of central or electric heating, non-owner, unemployment, foreign nationality, no access to a car, unskilled worker, household with more than six persons, low level of education, single-parent household). This index has been constructed from a European survey specifically designed to study deprivation and is composed of ecological variables identified to best reflect individual experience of deprivation. The ability of the EDI to measure individual deprivation was assessed in a preliminary validation [21] showing that scores were strongly associated with two individual socioeconomic variables, income and education level.

EDI was collected in 2011 at census block level to measure the socioeconomic deprivation of the areas of residence of the children’s families (higher index values indicating higher deprivation). To investigate the association between the EDI and CP, prevalence of CP and clinical characteristics were compared in three groups defined by the tertiles of the distribution of the EDI in the population of the surveillance area. To better consider the population at risk, i.e. the 8-year-old children, the distribution of EDI was weighted for the number of 8-year-olds residing in each census block in 2011. Thus, the total population of 8-year-old children residing in the surveillance area was allocated to these three deprivation groups based on the deprivation index of their census block. The first tertile (T1) corresponded to the 33% of 8-year-old children living in the least deprived census blocks.

### Prevalence calculation

The prevalence of CP was estimated in 8-year-old children over the entire period (2008–2019) in each census block group defined by EDI tertile. The denominator are the children aged 8 in each census unit between 2008 and 2019. Since the number of 8-year-olds at the census block level was not available for each year, the 2011 figure was taken and multiplied by 12 to cover the entire period of 12 years. Thus, in each tertile, prevalence was calculated using the formula: total number of 8-year-old children with CP between 2008 and 2019 / number of children aged 8 in each census unit according to the 2011 census data x 12. Census data from 2011 corresponded to the middle of the studied period, which yielded an average population number considering the regular population increase in this area over this period. Prevalences were estimated for all CP, for CP with ID and for CP with inability to walk.

### Statistical analysis

The association between deprivation measured by EDI and CP prevalence and CP functional outcome was estimated without any adjustment as no confounding variable was identified (see Directed Acyclic Graph in S1 Appendix).

In order to evaluate the potential bias induced by non-adjustment on unmeasured confounders, we computed E-values for both the observed association estimates and the lower limit of the confidence intervals (or upper limit if association estimates are under 1) [22]. These values indicate the minimum strength of association that these unmeasured confounders would need to have with both ecological measure of deprivation and CP to fully explain the association between deprivation and CP (reduce the observed association or its Confidence Interval to null).

Prevalence risk ratios (PRR) were estimated to compare CP prevalence across the three tertiles of deprivation levels using negative binomial regression models to account for dependent variables with over-dispersion.

As complementary analyses, we focused on the clinical characteristics of the children with CP which were first compared between preterm and term born children using chi2 and Fisher’s exact tests. In order to better explore the differences between these two groups, a sensitivity analysis was performed by removing children with non-spastic forms of CP known to be predominant in children born at term and of more severe clinical phenotypes [23–25]. Then clinical characteristics were compared across deprivation tertiles using chi2 and Fisher’s exact tests.

As presented in the DAG, we assumed that deprivation had an impact on some functional prognosis (ID or motor skills) mediated through both the origin of CP and the care from birth to age 8. Because CP children born preterm differ from CP children born at term (brain lesions, immaturity of the brain) the description of the repartition of ID and motor skills across deprivation tertiles was stratified on the preterm/term born status.

Statistical significance was based on a p-value of < 0.05.

All analyses were performed using STATA/IC software (version 11.1; Stata Corp., College Station, TX, USA).

## Results

**Fig 1:**
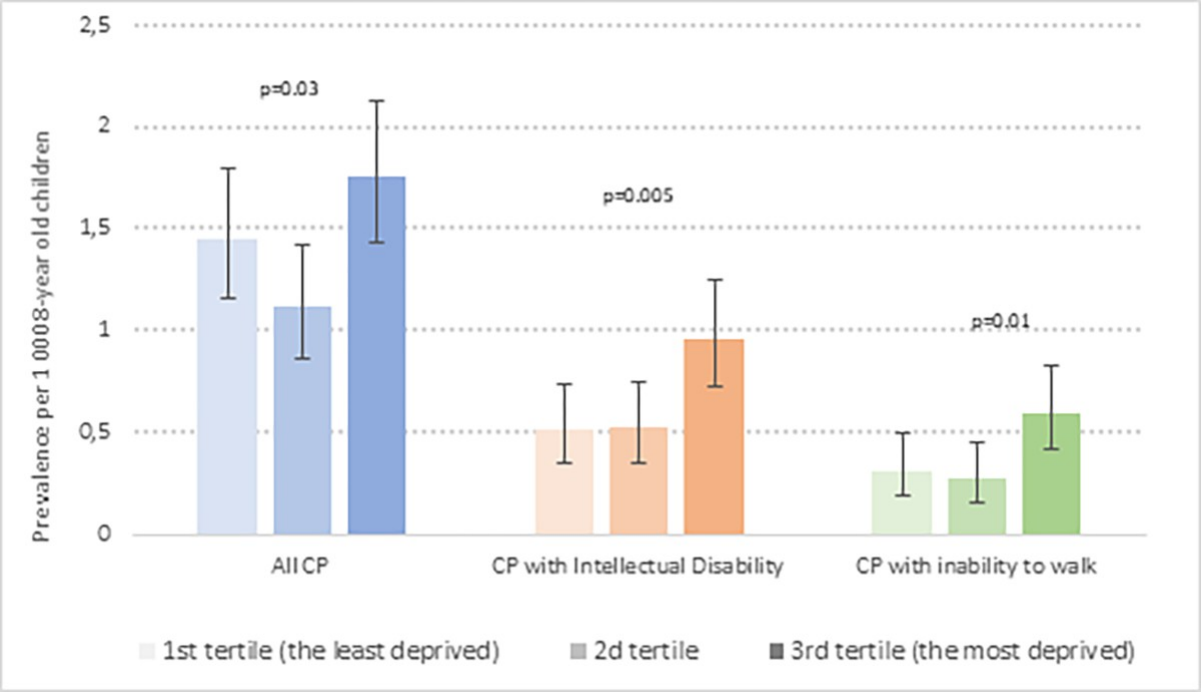
Prevalence and 95% Confidence Interval (CI) of CP (8-year old children born between 2000 and 2011) by three census block groups defined by distribution tertiles of the French European Deprivation Index, weighted on the number of 8-year-old children residing in each block. Prevalences are expressed for 1000 8-year children residing in same areas during the same period. Comparison of prevalence between three groups used negative binomial regression models (*p*-values).

The prevalence of CP in each of the three deprivation tertiles is presented in Fig 1 and the PRR comparing these prevalences across tertiles are presented in Table 1. The prevalence of all CPs significantly differed between tertiles (*p* = 0.03) with a **J**-shaped form. The prevalence in the most deprived tertile (T3) was the highest but not significantly different of the prevalence in the least deprived one (T1) (Table 1). However, the prevalence in the medium deprivation tertile (T2) was significantly lower than that in the most deprived one (PPR_*T2/T3*_ = 0.63 _*95% CI*_ [0.44–0.89]). The prevalence of CP associated with ID differed significantly between the three tertiles (*p* = 0.005, Fig 1) with a significant difference in prevalence between T3 versus T1 (PRR_*T3/T1*_ = 1.86 _*95% CI*_ [1.19–2.92]). Similarly, the prevalence of CP with no walking ability was significantly higher in the most deprived tertile compared to the least one (PRR_*T3/T1*_ = 1.90 _*95%CI*_ [1.07–3.37]).

**Table 1:** Prevalence risk ratio (PRR) and 95% Confidence Interval (negative binomial regression models) by deprivation risk group defined by distribution tertiles of the French EDI^ɣ^ weighted on the number of 8-year-old children residing in each block. The first tertile (T1) is the least deprived and the 3^rd^ tertile is the most deprived (T1 used as reference). Birth years 2000 to 2011.

	T2 vs T1		T3 vs T1	
	PRR^†^	95%CI^‡^	PRR^†^	95%CI^‡^
All CP	0.75	[0.52–1.08]	1.20	[0.87–1.66]
CP with intellectual disabilities	1.01	[0.61–1.70]	1.86**	[1.19–2.92]
CP with inability to walk	0.84	[0.42–1.68]	1.90*	[1.07–3.37]

^†^ PRR, prevalence risk ratio.

^‡^CI, confidence interval.

^ɣ^ EDI, European Deprivation Index (French version).

* p< 0.05.

** *p*<0.01.

For statistically significant estimates, the E-values were as follows: for prevalence of all CPs, the E-value of the PPR_*T2/T3*_ is 2.55 and the E-value of the upper limit of CI is 1.50. For the prevalence of CP associated with ID, the E-value of the PRR_*T3/T1*_ is 3.12 and the E-value for the lower limit of the CI is 1.67. For the prevalence of CP with no walking ability, the E-value of the PRR_*T3/T1*_ is 3.21 and the E-value for the lower limit of the CI is 1.34.

The following results concern the study of characteristics of the CP children.

Table 2 describes the clinical characteristics, the indicators of severity and the associated disorders in both preterm and term groups. Information was missing for 12 children on gestational age, for 2 children on BFMF and for 3 children on ID. The distribution of CP subtypes showed a clear predominance of bilateral (160 cases, 63.5%) and unilateral (50 cases, 19.8%) spastic forms compared to other non-spastic subtypes (dyskinetic (11 cases, 4.4%), ataxic (22 cases, 8.7%) or unclassifiable (9 cases, 3.6%) CP).

**Table 2:** Clinical characteristics, indicators of severity and associated disorders of children with CP born between 2000 and 2011 according to their term group.

	Total sample	Preterm group (<37weeks)	Term group (≥37weeks)	
	*n*/N (%)	*n*/N (%)	*n*/N (%)	p^¥^
Number of cases	252	112/240^a^ (46.7)	*128*/240 ^a^ (53.3)	
Male sex	*150*/252 (59.5)	*64*/112 (57.1)	*79/128* (61.7)	*ns*
Sub-types				**
CP type Spastic bilateral	*160/252* (63.5)	*91/112* (81.3)	*64/128* (50.0)	
CP type Spastic unilateral	*50/252* (19.8)	*14/112* (12.5)	*31/128* (24.2)	
CP type other	*42/252* (16.7)	*7/112* (6.3)	*33/128* (25.8)	
Inability to walk (GMFCS IV and V) ^†^	*69/252* (27.4)	*28/112* (25.0)	*39/128* (30.5)	*ns*
Moderate to severe BFMF (III to V) ^‡^	*56/250* (22.4)	*19/112* (17.0)	*35/126* (27.8)	*
Inability to walk AND moderate to severe BFMF	*45/251* (17.9)	*16/112* (14.3)	*28/127* (22.1)	*ns*
Intellectual disability (IQ<70)	*117/249* (47.0)	*37/110* (33.6)	*73/127* (57.5)	**
Epilepsy	*85/252* (33.7)	*26/112* (23.2)	*54/128* (42.2)	**
Visual or hearing impairment ^ɣ^	*113/252* (44.8)	*57/112* (50.9)	*52/128* (40.6)	*ns*
Associated malformation	*52/252 (20*.*6)*	*11/112 (9*.*8)*	*38/128 (29*.*7)*	**

^a^ Missing data on gestational age for 12 children with CP.

^¥^ *chi2 test*

* *p*<0.05

** *p*<0.01 ns: Non significant.

^£^ IQ, Intellectual Quotient

^†^ GMFCS, Gross Motor Function Classification System

^‡^ BFMF, Bimanual Fine Motor Function Classification.

^ɣ^ Visual or hearing impairment: Visual impairment (severe with acuity less than 3/10 or less severe with nystagmus or amblyopia or strabismus or other congenital anomalies) OR hearing impairment (regardless of severity).

Sub-type of CP, bimanual function and proportion of associated disorders differed between the two groups. Term-born children were more likely to have non-bilateral-spastic forms of CP compared to preterm children (*p*<0.01) and were more likely to have moderate to severely impaired bimanual fine motor function (*p*<0.05). They were significantly more likely to have ID (*p*<0.001) and epilepsy (*p*<0.01). They were also more likely to have some malformations (*p*<0.01). Gross motor function and sensorial disorders didn’t differ between preterm or term born children. MRI findings also differed significantly between preterm and term born children (p<0.001). The predominant white matter injuries concerned 74% of the children born prematurely versus 23% of the children born at term while maldevelopments and predominant grey matter injuries concerned respectively 5% for each category in children born preterm versus 23% and 16% for children born at term. Normal results were found respectively for 9.5% and 11% of preterm and term born children. After removing the non-spastic CP cases the difference between the preterm and term groups remained significant for the risk of associated intellectual disability (54.7% in term and 32.7% in preterm, p<0.01) and for the risk of associated epilepsy (41.1% and 22.9% respectively in term and preterm infants, p<0.01) whereas the difference concerning the severity of fine motor impairment was no longer significant (moderate to severe BFMF 26.9% and 18.1% respectively in term and preterm infants, p = 0.14).

Sample characteristics by deprivation tertiles risk groups level are shown in Table 3. No significant differences were found between deprivation level and sub-types of CP, maternal age at birth, proportion of preterm births (46.7% of the whole sample) or of low birthweights.

**Table 3:** Proportion of sub-types of cerebral palsy, maternal age at birth and perinatal characteristics of the children included (born 2000–2011) by census block groups defined by European Deprivation Index (EDI) distribution tertiles after weighting for the number of 8-year-old children residing in each block. The 1^st^ tertile (T1) corresponds to the least deprived.

	Total	T1 (least deprived) (%)	T2 (%)	T3 (%)	P*
Subtype of CP	*N = 252*	*N = 84*	*N = 64*	*N = 104*	*ns*
Spastic bilateral	63.5	60.7	65.6	64.4	
Spastic unilateral	19.8	25.0	17.2	17.3	
Other forms (dyskinetic, ataxic, unclassified)	16.7	14.3	17.2	18.3	
Maternal age at birth ^‡^	*N = 238* ^*‡*^	*N = 81*	*N = 60*	*N = 97*	
<25 years	9.7	7.4	5.0	14.4	*ns*
>38 years	9.2	8.6	8.3	10.3	*ns*
Preterm birth (<37 weeks) ^†^	*N = 240*^*†*^ 46.7	*N = 81* 48.2	*N = 63* 39.7	*N = 96* 50.0	*ns*
Low birthweight (<2500g) ^†^	*N = 236*^*†*^ 51.3	*N = 83* 54.2	*N = 62* 43.6	*N = 91* 53.9	*ns*

*Comparison between the 3 tertiles (chi2 test).

^‡^ Maternal age available for 238 children (14 missing data).

^†^ Gestational age available for 240 and birthweight for 236 of the 252 CP children.

ns: Non significant.

Table 4 shows the differences between deprivation risk groups and indicators of CP severity or associated disorders. For ID and motor impairments, results are presented separately for preterm and term-born children. Among children born prematurely the severity of CP and the presence of associated disorders significantly increased with increasing tertiles of deprivation. Compared to those living in the least deprived areas (T1), children born prematurely and living in T2 and in the most deprived areas (T3) were more likely to have severe forms of motor impairment such as an inability to walk (7.7%, 28.0% and 37.5%, *p*<0.01) or even very severe forms with a combination of an inability to walk and moderate to severe impairment of bimanual function (2.6%, 16.0% and 22.9%, *p*<0.05). They were also more likely to have associated intellectual disability (13.2%, 32.0% and 51.1%, *p*<0.01) compared to those living in the least deprived areas (T1).

**Table 4:** Proportions of indicators of severity and associated disorders by European Deprivation Index (EDI) deprivation risk groups distribution tertiles after weighting for the number of 8-year-old children residing in each block, among term and preterm children with CP. The 1^st^ tertile (T1) corresponds to the least deprived.

		Total Sample N = 252			Preterm born N = 112			Term born N = 128		
Outcome		%	95% CI ^β^	P^¥^	%	95% CI ^β^	p^¥^	%	95% CI ^β^	p^¥^
Inability to walk (GMFCS^†^ IV & V)		N = 252		*ns*	N = 112		**	N = 128		*ns*
	T1	21.4	[12.6–30.3]		7.7	[0–16.3]		33.3	[18.8–47.9]	
	T2	25.0	[14.3–35.7]		28.0	[9.8–46.2]		23.7	[9.9–37.5]	
	T3	33.7	[24.5–42.8]		37.5	[23.5–51.5]		33.3	[19.7–46.9]	
Moderate to severely impaired BFMF^‡^ (III to V)		N = 250		*ns*	N = 112		*ns*	N = 126		*ns*
	T1	20.2	[11.6–28.9]		7.7	[0–16.3]		28.6	[14.6–42.5]	
	T2	19.0	[9.2–28.9]		20.0	[3.8–36.2]		18.9	[6.0–31.8]	
	T3	26.2	[17.6–34.8]		22.9	[10.8–35.1]		34.0	[20.2–47.9]	
Inability to walk AND moderate to severe BFMF		N = 251		*ns*	N = 112		*	N = 127		*ns*
	T1	14.3	[6.7–21.9]		2.6	[0–7.6]		23.8	[10.6–37.0]	
	T2	14.3	[5.5–23.0]		16.0	[1.2–30.8]		13.5	[2.2–24.8]	
	T3	23.1	[14.9–31.3]		22.9	[10.8–35.1]		27.1	[14.3–39.9]	
Intellectual disability (ID) (defined as IQ^£^<70)		N = 249		*	N = 110		**	N = 127		*ns*
	T1	36.1	[25.7–46.6]		13.2	[2.1–24.2]		57.1	[41.8–72.4]	
	T2	47.6	[35.1–60.1]		32.0	[13.1–50.9]		56.8	[40.4–73.1]	
	T3	55.3	[45.6–65.0]		51.1	[36.5–65.7]		58.3	[44.1–72.6]	
Epilepsy		N = 252		*ns*						
	T1	31.0	[21.0–79.0]							
	T2	37.5	[25.5–49.5]							
	T3	33.7	[24.5–42.8]							
Associated visual or hearing impairment ^ɣ^		N = 252		*ns*						
	T1	38.1	[27.6–48.6]							
	T2	51.6	[39.2–64.0]							
	T3	46.2	[36.5–55.8]							

^¥^ *chi2 test*

* *p*<0.05

** *p*<0.01 ns: Non significant.

^β^ CI, confidence interval.

^£^ IQ, Intellectual Quotient

^†^ GMFCS, Gross Motor Function Classification System

^‡^ BFMF, Bimanual Fine Motor Function Classification.

^ɣ^ Visual or hearing impairment: Visual impairment (severe with acuity less than 3/10 or less severe with nystagmus or amblyopia or strabismus or other congenital anomalies) OR hearing impairment (regardless of severity).

To assess the extent to which these differences might be associated with the severity of prematurity, we performed a complementary analysis which separately considered the children born very preterm (64 children born before 32 weeks of gestation) and the moderate preterm children (48 children born at 32–36 weeks). Results (available in S1 Table) remained unchanged for ID in the two groups (p<0.05). For walking disability, for the combination of walking disability and moderate to severe impairment of bimanual function, a similar pattern of increasing severity with increasing tertile of deprivation was observed, but these results were no longer significant.

None of these associations were observed among term-born children. Epilepsy, visual and hearing impairment were not associated with deprivation.

## Discussion

To our knowledge, this is the first French population-based study to analyse the association between socioeconomic deprivation and the prevalence and characteristics of CP. Prevalence of pre-perinatal CP differed across the risk group of deprivation with a **J**-shaped form with a highest prevalence observed in the most deprived group. Even if we didn’t observe any significant difference between T1 and T3, the prevalence in the medium deprivation tertile (T2) was significantly lower than that in the most deprived one. Moreover, we observed that the prevalence of CP with associated ID and of CP with inability to walk was significantly higher in the most deprived tertile (T3) compared to the least deprived one (T1). Children born preterm and living in the most deprived areas were more likely to have severe forms of motor impairment such as an inability to walk or even very severe forms with a combination of an inability to walk and moderate to severe impairment of bimanual function and they were also more likely to have associated ID.

In our study, we did not significantly demonstrate a clear gradient (i.e an increase with each increasing tertile of deprivation) in the prevalence of CP as a whole, but only for those forms with intellectual disability or severe functional impairment. Some previous publications [1,3,5,6] have reported a deprivation gradient in CP prevalence. When such a gradient is observed, it can be mediated by higher risk of preterm birth or low birthweight among children living in the most deprived areas, [4] although some studies underline an independent residual effect of deprivation [3,5] or maternal educatio n [8] on CP prevalence. In the general French population as elsewhere, prematurity and low birthweight are more common among more deprived families, [26,27] with a continuous gradient across deprivation area previously described in a French survey in the Ile-de-France region [28]. If there was an impact of deprivation on CP prevalence completely mediated through prematurity, we should have observed the same continuous deprivation gradient for CP prevalence which is not the case, even though prevalence tends to be higher in the most deprived tertile. Furthermore, in our sample, the proportion of children born preterm did not increase with each increasing tertile of deprivation unlike what is usually observed in the general population (Table 3). Similar results were found in recent publication [13] and in the publication of Dolk et al. [14] where the proportion of low-birthweight CP children was lowest in the most deprived area despite the highest proportion of low-birthweight births in these areas in the general population. CP risk factors are known to differ according to gestational age, [29,30] and among preterm children the risk may be associated, at least partly, with the cause of prematurity. Among preterm children, preeclampsia and intrauterine growth retardation may represent a lower CP risk than other causes of prematurity. Moreover, the impact of deprivation on the risk of prematurity involves complex mechanisms [31]. Deprivation may lead to a large range of causes of prematurity which could each carry, in turn, very different and potentially opposite risks for developing CP. Similarly, Dolk et al. [14] suggested that “the causes of moderate or low birth weight in the more deprived areas (….) may be associated with lower CP risk than other risk factors that reduce birthweight”. These paradoxical effects are also commented by Solaski et al. [4].

We found that prevalence was significantly higher in the most deprived areas for CP associated with ID and for children who were unable to walk, consistently with previous publications on prevalence of CP with severe motor limitations [1,2,12]. These findings on prevalence are supported by the complementary descriptive analysis of the repartition of functional outcomes across deprivation tertiles.

As expected, our results confirm important differences in the clinical description of children with CP between term and preterm. As found in the literature, children born preterm present fewer non-spastic forms [24,25,32], mainly present predominant white matter injuries [17,18], and present fewer malformations than children born at term [33]. The differences observed concerning a higher frequency of severe fine motor impairment, intellectual disability and epilepsy in term CP children compared to preterm CP children are also found in the literature [32,34–36]. Sensitivity analysis based on the exclusion of children with non-spastic forms did not alter the differences found between preterm and term-born children with respect to intellectual disability and epilepsy. These results are consistent with those from studies which specifically focused on children with spastic forms of CP [32,34,35]. The hypotheses put forward for these differences between preterm and term-born children with the same (spastic) forms are notably that of a different cerebral vulnerability [32] and brain’s ability to compensate better when it is less well developed [34]. However, some studies have shown inconclusive [37] or contradictory results [25]. Other studies focused on children with the same type of brain lesions [38,39] have shown clinical differences between preterm and term born children with a greater severity of motor impairment in preterm children, leading the authors to suggest that "despite a common radiologic pattern, these are different clinicopathologic entities." Thus, although sometimes contradictory in the literature, these results clearly highlight the difference in type of CP, underlying lesions, and clinical presentation between preterm and term infants.

Our results demonstrated that repartition of functional outcomes across deprivation tertiles clearly differed between preterm and term-born children.

Proportion of preterm CP children with inability to walk, combination of an inability to walk and moderate to severe impairment of bimanual function or with associated ID was higher among those living in the most deprived areas while no difference was observed among term-born CP children. Few studies have analysed these associations separately in preterm and term-born CP children. Maenner et al. [12] observed that severe forms of CP were more prevalent in black children whatever their term group. Recently and more precisely, Woolfenden et al. [13] showed that socio-economic disadvantage at birth impacts adversely on CP severity both in premature and term-born infants. But conversely and in a similar way to what we find in our study, Oskoui et al [10] found a socio-economic gradient for non-ambulant children only in preterm CP children.

We might assume that the proportion of extreme and severe prematurity would probably be higher among the most deprived preterm children compared to the less deprived preterm children. However, when the analyses are separated according to the degree of prematurity (<32 weeks vs 33–36), and although the small numbers make interpretation difficult, the results are nevertheless unchanged for intellectual disability and the trends remained similar in the two groups, although not significant, for motor disorders. Similarly, in a French cohort of very preterm children (Epipage2), a deprivation gradient was shown in severe neonatal morbidity even after controlling for gestational age [27].

The deprivation gradient observed among preterm children with CP is consistent with many previous results on very preterm children in general. Very preterm children born to parents with low socioeconomic status are known to be at increased risk of cognitive deficiency, even after considering cerebral lesions and several other medical conditions [40–42]. It has been suggested that the effect of deprivation may be mediated by delayed access or even lack of access to rehabilitation care [43], which may influence the motor and cognitive prognosis of CP. Despite a universal healthcare system supposed to offer appropriate care to each child, inequality in awareness and in access to appropriate care may be a reality. It seems very likely that the most privileged families may be more aware of the developmental difficulties of their child, more knowledgeable about the care system and, finally, more able to turn towards private specialised care that is not provided by the national health insurance system. It seems plausible that the least deprived families may benefit from earlier and more appropriate care, although the underlying effect appears complex and not easily measurable. The other possible mechanism is a link between deprivation and the causes of CP in children born preterm, thus potentially a link between deprivation and some causes of prematurity that would generate more severe forms of CP than others. In our study, the lack of data on the cause of prematurity or the direct cause of CP makes it difficult to further investigate this hypothesis.

On the other hand, no association between deprivation and functional outcome was observed among children with CP born at term. As we have seen, one of the reasons may be that the causal pathways of CP differ in preterm and in term-born children, resulting in different clinical presentations [44,45]. As found in our sample, term-born children appear to have different types of CP, different cerebral lesions, they are clearly at higher risk of ID and epilepsy and in previous work, we had found that they were also at greater risk of autism spectrum disorders [46], all of this leading to more severe developmental forms of CP. These more complex forms with cerebral lesions much more varied and extended to the grey matter and which occur on more mature brains may be less responsive to the benefits of early management on cognitive or motor development, and so the underlying effects of deprivation on an earlier and more appropriate access to care may be less visible in this group.

In conclusion, while El Hassan et al. [47] showed that, in the general population, the effect of socioeconomic level on educational outcomes did not differ between gestational groups, these effects appeared different in children with CP, possibly because of the particular severity of the associated cognitive disorders in term-born infants.

However, all these hypotheses cannot be entirely supported by our results because of the limits of the descriptive analysis performed among CP children. Firstly, we were not able to evaluate how the rehabilitation mediates the path between deprivation and functional outcome, as we don’t have any information which could describe all the care and rehabilitation that the children actually benefited from. Secondly, we don’t have the data concerning the intermediate factors occurring before birth, so that we were not able to decompose the causal paths implicated. Finally, we assumed that the impact of care from birth to age 8 could be different between preterm and term born children meaning that preterm/term born status could act both as a mediating and an interaction factor between deprivation and functional outcome. As a consequence, results are presented as an exploratory analysis of the direct effect of deprivation which cannot fully lead to a formal causal interpretation of the associations described because of the lack of complementary variables necessary to properly adjust on, distinguish and decompose all the causal path.

The main strength of this study is the use of a population-based CP register which ensures a reliable and stable over time definition of CP. Moreover, the use of an area measure of deprivation prevents non-random missing data. One of the limitations is that the area deprivation measures were related to the parental address at the age of inclusion in the register and not at birth. Firstly, this prevents any formal conclusion on a causal relationship between deprivation and the prevalence or characteristics of CP. Secondly, parents may have moved during the time between their child’s birth to the age of 8. This could limit the interpretation of the results only if families had moved to a different deprivation-tertile census block more often than the general population used as denominator. Dolk et al. [14] showed that there was no obvious indication that children with CP were more likely to move into affluent areas than other children. Another limitation concerns changes in deprivation in the general population as a whole over the study period. However, this shouldn’t bias the results since we don’t have any argument to suspect that the impact of deprivation on CP could change over time. Also, we must be cautious about the possibility of extrapolating the data to the entire French national territory because of the limited geographic coverage of the register. Finally, sensitivity analyses have been performed by computing E-values to evaluate the potential bias induced by non-adjustment on unmeasured confounders on the associations between deprivation measured by EDI and CP prevalence. The only source of confounding that we could hypothesize was confounding by the individual (unmeasured) socioeconomic characteristics of the families which could both impact ecological measure of deprivation and CP occurrence. They would thus be the only unmeasured factors that would bias the measure of the total effect only of the environmental dimension of deprivation on CP occurrence. E-values would mean that the association between the contextual effect of deprivation and CP prevalence would be reduced to null if the individual effect of deprivation reached an RR of 2.55 or 3. However, even if EDI remains an ecological measure, it has been constructed to best reflect the individual experience of deprivation. And since individual socio-economic characteristics of the families were not available, the individual and contextual effects of deprivation cannot be disentangled, as mentioned in the discussion of a recent article on the robustness of the EDI indicator [48]. This reinforces the fact that deprivation as measured in our study should be interpreted as a multi-level dimension, a mix between individual and environmental aspects of socio-economic level.

Our study adds some findings that are consistent with results already observed, and it highlights in particular the difference in the impact of deprivation between preterm and term-born children with CP. Children with CP born preterm and living in the most deprived areas showed a higher burden of ID and impaired motor function. Further studies should deeper assess the interactions between a deprived environment and prematurity on developmental prognosis for children with CP.

## Supporting information

S1 Table Complementary analysis for children born preterm separately for children born very preterm (before 32 weeks of gestation) and moderate preterm (born at 32–36 weeks) for indicators of severity significantly associated with deprivation among preterm children (table IV). Proportions of indicators of severity and associated disorders by European Deprivation Index (EDI) deprivation risk groups distribution tertiles after weighting for the number of 8-year-old children residing in each block, among very preterm term and moderate preterm children with CP. The 1^st^ tertile (T1) corresponds to the least deprived.
(DOCX)

S1 Appendix Directed acyclic graph (DAG). The directed acyclic graph (DAG) describes the hypothesized causal relationship between deprivation (exposure) and CP (outcome). Two kinds of pathways are represented: A) [in yellow]: A link between deprivation and the origin of CP which could impact both the occurrence and the severity and B) [in blue]: A path between deprivation and access to some intensive, ultra-early medical and re-educational care modalities which could impact the functional prognosis of some associated disorders such as intellectual disability or motor skills. Shaded text represents unmeasured factors. Green text represents unmeasured potential confounder. Preterm/Term born status [text colored in yellow] is supposed to act as a mediating (A) and interaction factor on (B).
(PDF)
