# Supplementary material for: Prevalence and characteristics of children with cerebral palsy according to socioeconomic status of areas of residence in a French department
Source: PLoS One. 2022 May 19;17(5):e0268108. doi: 10.1371/journal.pone.0268108 (PMC9119545; doi:10.1371/journal.pone.0268108)
Supplement: S1 Table — Proportions of indicators of severity and associated disorders by European Deprivation Index (EDI) deprivation risk groups distribution tertiles after weighting for the number of 8-year-old children residing in each block, among very preterm term and moderate preterm children with CP. The 1st tertile (T1) corresponds to the least deprived. (DOCX) [file pone.0268108.s001.docx]

**S1 Table:** Complementary analysis for children born preterm separately for children born very preterm (before 32 weeks of gestation) and moderate preterm (born at 32-36 weeks) for indicators of severity significantly associated with deprivation among preterm children (table IV). Proportions of indicators of severity and associated disorders by European Deprivation Index (EDI) deprivation risk groups distribution tertiles after weighting for the number of 8-year-old children residing in each block, among very preterm term and moderate preterm children with CP. The 1^st^ tertile (T1) corresponds to the least deprived.

|  |  | Very Preterm born N=64 | | | Moderate preterm born N=48 | | |
| --- | --- | --- | --- | --- | --- | --- | --- |
| Outcome |  | % | 95% CI ^β^ | p^¥^ | % | 95% CI ^β^ | p^¥^ |
| Inability to walk (GMFCS^†^ IV & V) | | N=64 | | *ns* | N=48 | | *** |
|  | T1 | 13.0 | [4.1-34.7] |  | 0 |  |  |
|  | T2 | 42.9 | [19.7-69.7] |  | 9.1 | [1.1-47.8] |  |
|  | T3 | 40.7 | [23.6-60.4] |  | 33.3 | [16.1-56.5] |  |
| Inability to walk AND moderate to severe BFMF ^‡^ | | N=64 | | *ns* | N=48 | | *ns* |
|  | T1 | 4.3 | [0.6-26.9] |  | 0 |  |  |
|  | T2 | 21.4 | [6.6-51.3] |  | 9.1 | [1.1-47.8] |  |
|  | T3 | 29.6 | [15.1-49.8] |  | 14.3 | [4.4-37.6] |  |
| Intellectual disability (ID) (defined as IQ^£^<70) | | N=64 | | *** | N=46 | | *** |
|  | T1 | 13.0 | [4.1-34.7] |  | 13.3 | [3.1-42.8] |  |
|  | T2 | 35.7 | [14.9-63.9] |  | 27.3 | [8.2-61.0] |  |
|  | T3 | 48.1 | [29.8-67.0] |  | 55.0 | [32.6-75.6] |  |

^¥^ *test exact*  * *p*<0.05 ns : non significant

^β^ CI. confidence interval

^†^ GMFCS. Gross Motor Function Classification System

^‡^ BFMF. Bimanual Fine Motor Function Classification

^£^ IQ. Intellectual Quotient
