# Supplementary material for: Prevalence and characteristics of children with cerebral palsy according to socioeconomic status of areas of residence in a French department
Source: PLoS One. 2022 May 19;17(5):e0268108. doi: 10.1371/journal.pone.0268108 (PMC9119545; doi:10.1371/journal.pone.0268108)
Supplement: S1 Appendix — The directed acyclic graph (DAG) describes the hypothesized causal relationship between deprivation (exposure) and CP (outcome). Two kinds of pathways are represented: A) [in yellow]: A link between deprivation and the origin of CP which could impact both the occurrence and the severity and B) [in blue]: A path between deprivation and access to some intensive, ultra-early medical and re-educational care modalities which could impact the functional prognosis of some associated disorders such as intellectual disability or motor skills. Shaded text represents unmeasured factors. Green text represents unmeasured potential confounder. Preterm/Term born status [text colored in yellow] is supposed to act as a mediating (A) and interaction factor on (B). (PDF) [file pone.0268108.s002.pdf]

S1 Appendix

Directed acyclic graph (DAG)

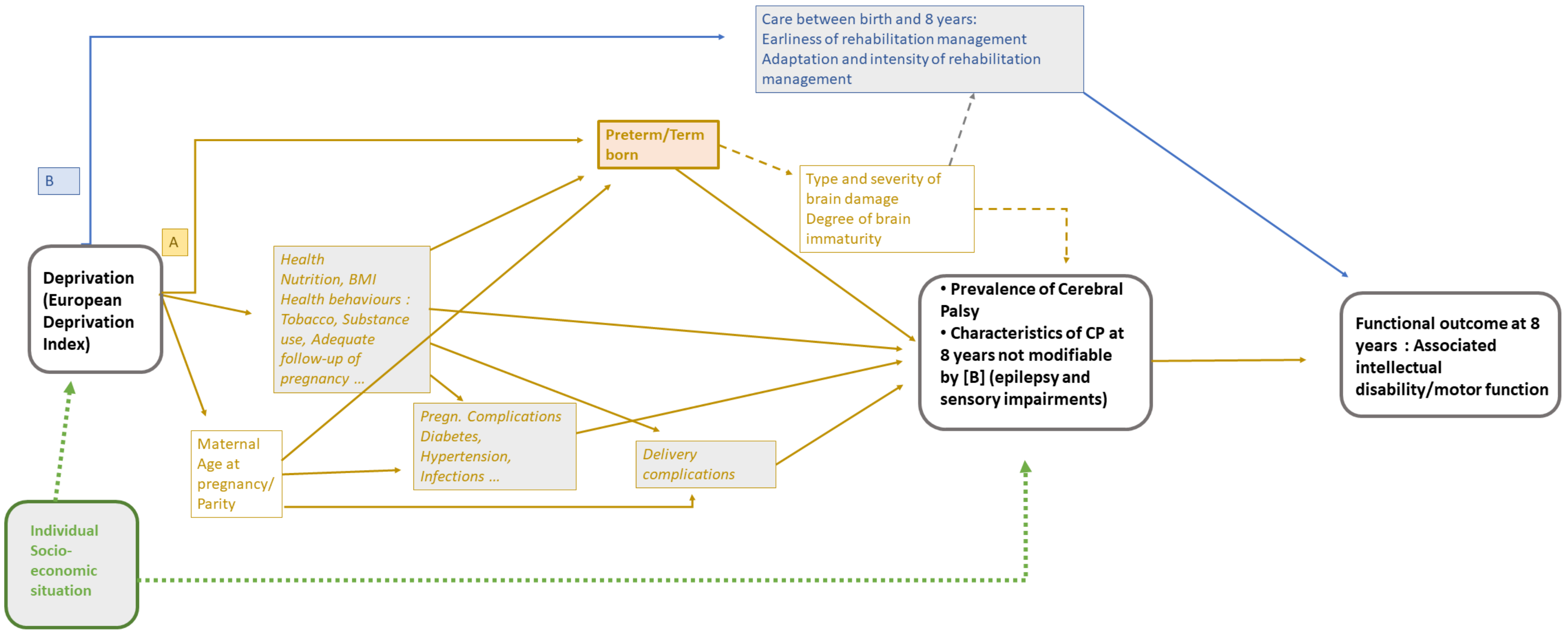

The directed acyclic graph (DAG) describes the hypothesized causal relationship between deprivation (exposure) and CP (outcome).
Two kinds of pathways are represented: A) [in yellow] : a link between deprivation and the origin of CP which could impact both the occurrence and the severity and B) [in blue] : a path between deprivation and access to some intensive, ultra-early medical and re-educational care modalities which could impact the functional prognosis of some associated disorders such as intellectual disability or motor skills.
Shaded text represents unmeasured factors. Green text represents unmeasured potential confounder.
Preterm/Term born status [text colored in yellow] is supposed to act as a mediating (A) and interaction factor on (B)
